# Boosting CAR T-cell responses in lymphoma by simultaneous targeting of CD40/4-1BB using oncolytic viral gene therapy

**DOI:** 10.1007/s00262-021-02895-7

**Published:** 2021-03-05

**Authors:** Jessica Wenthe, Sedigheh Naseri, Alireza Labani-Motlagh, Gunilla Enblad, Kristina I. Wikström, Emma Eriksson, Angelica Loskog, Tanja Lövgren

**Affiliations:** 1 Science for Life Laboratory, Department of Immunology, Genetics and Pathology, Uppsala University, Rudbeck Laboratory C11, Dag Hammarskjölds Väg 20, 75185 Uppsala, Sweden; 2 Cell Therapy Center, Vecura, Karolinska University Hospital, Stockholm, Sweden; 3 Lokon Pharma AB, Uppsala, Sweden

**Keywords:** B-cell lymphoma, CAR T cells, Oncolytic virus, CD40L, 4-1BBL

## Abstract

Pretreatment of B-cell lymphoma patients with immunostimulatory gene therapy using armed oncolytic viruses may prime tumor lesions for subsequent chimeric antigen receptor (CAR) T-cell therapy, thereby enhancing CAR T-cell functionality and possibly increasing response rates in patients. LOAd703 (delolimogene mupadenorepvec) is an oncolytic adenovirus (serotype 5/35) that encodes for the transgenes CD40L and 4-1BBL, which activate both antigen-presenting cells and T cells. Many adenoviruses failed to demonstrate efficacy in B-cell malignancies, but LOAd703 infect cells via CD46, which enables B cell infection. Herein, we investigated the therapeutic potential of LOAd703 in human B-cell lymphoma models, alone or in combination with CAR T-cell therapy. LOAd703 could infect and replicate in B-cell lymphoma cell lines (BC-3, Karpas422, Daudi, DG-75, U-698) and induced an overall enhanced immunogenic profile with upregulation of co-stimulatory molecules CD80, CD86, CD70, MHC molecules, death receptor Fas and adhesion molecule ICAM-1. Further, CAR T-cell functionality was boosted by stimulation with lymphoma cells infected with LOAd703. This was demonstrated by an augmented release of IFN-γ and granzyme B, increased expression of the degranulation marker CD107a, fewer PD-1 + TIM-3+ CAR T cells in vitro and enhanced lymphoma cell killing both in in vitro and in vivo xenograft models. In addition, LOAd703-infected lymphoma cells upregulated the secretion of several chemokines (CXCL10, CCL17, CCL22, CCL3, CCL4) essential for immune cell homing, leading to enhanced CAR T-cell migration. In conclusion, immunostimulatory LOAd703 therapy is an intriguing approach to induce anti-lymphoma immune responses and to improve CAR T-cell therapy in B-cell lymphoma.

**Supplementary Information:**

The online version contains supplementary material available at 10.1007/s00262-021-02895-7.

## Introduction

B-cell lymphoma is among the most prevalent hematologic malignancies and is a heterogeneous disease with many subtypes based on tumor cell characteristics and pathogenesis [1]. Many patients relapse or are refractory to primary treatment with rituximab in combination with chemotherapy, and these patients have a poor prognosis [2, 3]. Chimeric antigen receptor (CAR) T-cell therapy targeting CD19^+^ B cells has recently been approved for treatment of diffuse large B-cell lymphoma after demonstrating improved outcomes in relapsed/refractory patients. However, initial response rates in lymphoma are considerably lower than previously noted in leukemia [4, 5]. This is likely due to physical barriers and immunosuppression in the solid lymphoma lesions [6]. Novel treatment approaches intending to modulate the tumor microenvironment (TME), such as immunostimulatory gene therapy using oncolytic viruses, are needed to further improve response rates and ultimately prolong survival.

The Lokon oncolytic adenovirus (LOAd) platform consists of serotype 5 adenoviruses with the fiber and knob from serotype 35. This modification retargets the viruses to infect CD46^+^ cells, thereby enabling infection of most cells, including B cells. Viral replication is restricted to cells with a dysregulated retinoblastoma pathway, causing selective lysis of cancer cells. Further, LOAd viruses are modified to encode immunostimulatory transgenes under a strong CMV promoter inducing expression in all infected cells independently of replication. LOAd703 (delolimogene mupadenorepvec), which is currently being evaluated in clinical trials for the treatment of melanoma, pancreatic, ovarian, colorectal and biliary cancer (NCT02705196, NCT03225989, NCT04123470), encodes for a trimerized membrane-bound (TMZ)-CD40L, and 4-1BBL [7]. These transgenes may be of particular interest for B-cell lymphoma and the combination with CAR T-cell therapy. The lymphoma cells originate from B cells, which are professional antigen-presenting cells, but they often harbor defects in their antigen presentation function, which enables escape from the immune system [8]. As CD40/CD40L is an essential signaling pathway in B cells, which promotes B cell maturation and activation [9], stimulation of malignant B cells with a CD40L-encoding adenovirus has been shown to result in improved ability to present antigens and activate cytotoxic T cells [10]. Moreover, stimulation of T cells by 4-1BBL is known to enhance their proliferation, effector function and survival, which may ultimately promote a CAR T-cell response and persistence [11–14]. In addition, LOAd703 has been shown to upregulate adhesion molecules and chemokines involved in lymphocyte migration, induce maturation of dendritic cells, increase antigen-specific T-cell frequency, and stimulate anti-tumor immunity and tumor elimination in vivo [7].

Herein, we are presenting the results from the preclinical assessment evaluating LOAd703 in B-cell lymphoma cell lines for its oncolytic effect and potential to induce an immunogenic phenotype. Furthermore, we explored if priming lymphoma cells with LOAd703 may enhance the efficacy of subsequent CAR T-cell therapy.

## Material and methods

### Cells

Human B-cell lymphoma cell lines BC-3 (RRID:CVCL_1080) and Daudi (RRID:CVCL_0008) were purchased from ATCC (Manassas, VA, USA) and Karpas422 (RRID:CVCL_1325), and DG-75 (RRID:CVCL_0244) and U-698 (RRID:CVCL_0017) were a gift from Prof. Nilsson (Uppsala University). The cell lines were tested for mycoplasma with MycoAlert™ Mycoplasma Detection Kit (Lonza, Basel, Switzerland). CAR T cells expressing a third generation CAR targeting CD19 and containing both CD28 and 4-1BB co-stimulatory domains were obtained from CAR T-cell batches produced for our previous clinical trial [15] and are herein analyzed as part of their functionality testing. The clinical trial had all needed approvals from the Uppsala regional ethical review board and the Medical Products Agency in Sweden. All patients signed informed consent. Cells were cultured in RPMI-1640 supplemented with penicillin (100 U/mL)/streptomycin (100 µg/mL) and 10% fetal bovine serum (FBS). Media and supplements were purchased from ThermoFisher Scientific (Waltham, MA, USA). CAR T cells were thawed and cultured in medium supplemented with 100 IU/mL of IL-2 (Proleukin®, Novartis) for 48 h prior to experiments.

### LOAd viruses

LOAd viruses were provided by Lokon Pharma, AB, Uppsala, Sweden. The generation of these viruses was described previously [16]. LOAd703 encodes immunostimulatory transgenes (TMZ-CD40L & 4-1BBL), which are expressed under the control of a CMV promoter [7, 16]. LOAd(-), lacking the transgene cassette, and a replication-deficient Mock adenovirus 5/35 were used as controls. For infection, cells were washed in serum-free medium and incubated with virus (multiplicity of infection (MOI): 10–200 fluorescence-forming units/cell) for 2 h, followed by addition of complete medium.

### Viability assay

Viability was analyzed with CellTiter 96 AQueous One Solution MTS reagent (Promega, Fitchburg, WI, USA). 1 × 10^4^ cells/well were plated in quadruplicates and cultured for 72, 96, 120 or 144 h before viability analysis.

### Quantification of viral DNA

Infected cells (50 MOI) were cultured for 2, 24, 48 and 96 h (2 × 10^5^ cells/well) before viral DNA was isolated with High Pure Viral Nucleic Acid kit (Roche, Basel, Switzerland) and quantified with primers detecting the adenoviral E4 orf1 transcript (Fw-5′CATCAGGTTGATTCACATCGG; Rw-5′GAAGCGCTGTATGTTGTTCTG) [17]. Quantitative PCR was performed using iQ SYBR® Green PCR Supermix and CFX96 Real-Time detection system (Bio-Rad, Hercules, CA, USA).

### Western blot

Cells were lysed with RIPA buffer containing Pefabloc (Sigma-Aldrich, Saint Louis, MO, USA). Lysates (100 µg) were mixed with sample buffer, denatured and loaded in Mini-Protean TGX precast gel (Bio-Rad) for SDS-PAGE and transferred to PVDF membranes (GE Healthcare, Chicago, IL, USA). Membranes were blocked with 3% ECL prime blocking agent (GE Healthcare) before primary antibodies (1:1000) against retinoblastoma protein (Rb (D20, Cat#9313S) or Phospho-Rb (Ser-795, Cat#9301S), Cell Signaling Technology, Danvers, MA, USA) were added. Membranes were washed with phosphate buffer saline (PBS)/0.05% Tween 20 before anti-rabbit IgG (1:9000, Cat#ab97051, Abcam, Cambridge, UK) was added. ECL prime western blotting detection reagents (GE Healthcare) were used for development, and bands were detected by ChemiDoc Touch Imaging system and analyzed with Image Lab 6 (Bio-Rad).

### Evaluation of functional viral particles

Culture supernatants of infected cells (50 MOI) were collected 48 h post infection, mixed 1:1 with fresh medium and added to plated Panc01 cells (1 × 10^4^ cells/well). Viability of Panc01 cells was measured 96 h later.

### Phenotype analysis

Infected cells (100 MOI) were cultured for 48 h, then washed in PBS supplemented with 3 mM EDTA (Thermo Fisher) and 0.5% bovine serum albumin (Sigma-Aldrich) and stained with fluorescent-labeled antibodies (BioLegend, San Diego, CA, USA) against CD40L (Clone:24–31, RRID:AB_2562721), 4-1BBL (Clone:5F4, RRID:AB_314883), CD95 (Clone:DX2, RRID:AB_314544), CD80 (Clone:2D10, RRID:AB_2076147), CD86 (Clone:IT2.2, RRID:AB_11204252), CD54 (Clone:HCD54, RRID:AB_2121926), HLA-DR (Clone:L243, RRID:AB_314688), HLA-A/B/C (Clone:W6/32, RRID:AB_314873), CD70 (Clone:113–16, RRID:AB_2561431), CD19 (Clone:HIB19, RRID:AB_314238) and CD46 (Clone:TRA-2–10, RRID:AB_10895756). Stained cells were fixed in PBS containing 1% formaldehyde and 3 mM EDTA (fixation buffer). Samples were analyzed with BD FACS Canto 2 (BD Biosciences, San Jose, CA, USA) and FlowJo software (RRID:SCR_008520, FlowJo LLC, Ashland, OR, USA).

### Combination with CAR T cells

Infected target cells (100 MOI), Karpas422 and DG-75, were cultured for 48 h before CAR T cell co-culture. For killing assays, target cells (5 × 10^4^/well) were plated in triplicates. CAR T cells were added to achieve an effector/target cell ratio of 10:1–0.3:1 dependent on the repeat. Culture supernatants were taken 48 h later and analyzed for lactate dehydrogenase (LDH) release with LDH-Glo™ Cytotoxicity Assay (Promega). Granzyme B and IFN-γ levels were quantified with ELISA development kits from Mabtech (Nacka Strand, Sweden). For flow cytometry analysis of CAR T cells, target and effector cells were cultured in a 1:1 ratio for 24 h. Cells were washed in PBS and stained with the dead cell marker Zombie NIR and fluorescent-labeled antibodies from BioLegend against CD3 (Clone:UCHT1, RRID:AB_893298), CD8 (Clone:RPA-T8, RRID:AB_314124), CD107a (Clone:H4A3, RRID:AB_2562648), CD69 (Clone:FN50, RRID:AB_2563834), 4-1BB (Clone:4B4-1, RRID:AB_2563830), PD-1 (Clone:EH12.2H7, RRID:AB_2159324), and TIM-3 (Clone:F38-2E2, RRID:AB_11218598). CAR scFv^+^ cells were detected with F(ab')_2_ Fragment antibody conjugated to DyLight 649 (Jackson ImmunoResearch, Cambridgeshire, UK). Stained cells were fixed and analyzed with BD FACS Canto 2 and FlowJo software.

### Migration assay

Culture supernatants were taken from Karpas422 and DG-75 cells 48 h post infection (100 MOI) and 600 µl were placed in the lower chamber of a 5.0 µm transwell (Corning, New York, USA). As negative and positive controls, fresh medium and medium supplemented with 250 ng/mL CXCL10 (BioLegend) were used, respectively. 1 × 10^6^ CAR T cells were placed in the upper chamber. After 6 h, cells in the lower chamber were counted with TC20 automated cell counter (Bio-Rad). Culture supernatants were analyzed with human V-PLEX Chemokine Panel 1 (Meso Scale Discovery, Rockville, MD, USA) according to manufacturer’s protocol.

### Xenograft model

Animal experiments were approved by the local animal ethical review board in Uppsala, Sweden (DNr 5.8.18-13,471/2017). DG-75 cells were mixed 1:1 with Corning Matrigel matrix (Corning, NY, USA) and injected subcutaneously in female immunodeficient BALB/c nude mice (2.5 × 10^6^ cells/mouse; 7 mice per group). Treatments were initiated at day 10, and mice received in total 6 intratumoral treatments with LOAd703 (1 × 10^9^ FFU/mouse) or saline as control. At the second treatment time point (day 13), CAR T cells were given intravenously (3.3 × 10^6^ cells/mouse). Tumor growth was monitored by measuring the ellipsoid tumor volume and mice with tumors over 1000 mm^**3**^ were sacrificed.

### Statistical analysis

GraphPad Prism 8 (RRID:SCR_002798, San Diego, CA, USA) was used for statistical analysis. Data showing a representative experiment with only technical replicates was considered normally distributed, and one-way ANOVA followed by Dunnet’s or Tukey’s multiple comparisons test was used to determine statistical differences compared to uninfected control group or all groups, respectively. Statistical analysis of data composed of several biological replicates was performed with Kruskal–Wallis test followed by Dunn’s multiple comparison test (*p < 0.05, **p < 0.01, ***p < 0.001, ****p < 0.0001).

## Results

### B-cell lymphoma cell lines are both targets for CAR T-cell and LOAd703 therapy

**Fig. 1:**
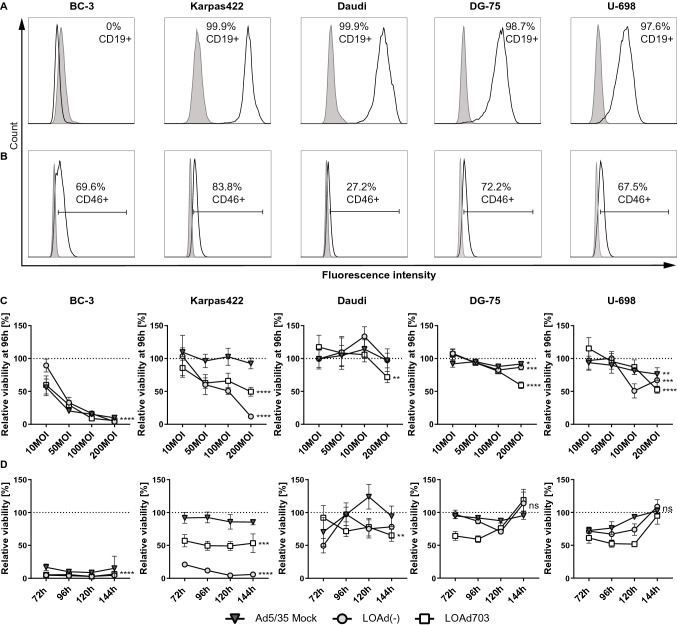
CD19 and CD46 expression in lymphoma cell lines and viability post LOAd infection. Human lymphoma cell lines (BC-3, Karpas422, Daudi, DG-75 and U-698) were analyzed for CD19 and CD46 expression by flow cytometry. Filled gray histograms display isotype control staining and overlaid black lines show CD19 (**a**) and CD46 expression (**b**). Lymphoma cells were infected with LOAd viruses or replication-deficient Ad5/35 Mock virus at different virus to cell ratios (10, 50, 100 and 200 MOI). Cell viability was analyzed with MTS viability assay 96 h post infection (**c**) and over an extended time period (72–144 h) for cells infected with 200 MOI (**d**). Viability is shown as percentage viability of uninfected control cells (mean ± SD of technical quadruplicates; representative of two independent experiments). Statistical differences compared to uninfected control cells were analyzed at 200 MOI (**c**) or 144 h (**d**) with one-way ANOVA followed by Dunnett’s multiple comparisons test (*p < 0.05, **p < 0.01, ***p < 0.001, ****p < 0.0001)

A panel of human B-cell lymphoma cell lines (BC-3, Karpas422, Daudi, DG-75, U-698) was selected for investigation. All cell lines, except for BC-3, expressed CD19 and can be targeted by CAR T-cells (Fig. 1a). As LOAd703 infects cells via CD46, we also determined CD46 expression of the cell lines. All cell lines were CD46+ and are therefore also good targets for LOAd703 therapy. However, CD46 expression varied between the cell lines with lower levels in Daudi (27.2%) compared to the other cells (67.5%–83.8%) (Fig. 1b). To assess the killing capability of LOAd703, lymphoma cells were infected at different MOI with LOAd703 and with LOAd(-) lacking the transgenes or with replication-deficient Mock virus as control (Fig. 1c). BC-3 was most susceptible to killing as shown by reduced viability at all MOIs. However, BC-3 was also sensitive to Mock virus, indicating that the killing was not only due to replication-mediated oncolysis but to viral infection per se. Karpas422 cells were specifically and efficiently killed by LOAd infection. Daudi, DG-75 and U-698 displayed only a modest reduction of viability at the highest MOI. To evaluate if this was due to a difference in kinetics, cells were infected with 200 MOI and cultured for up to 144 h (Fig. 1d). For BC-3, Karpas422 and Daudi, the drop in viability remained stable at 144 h compared to 96 h. In contrast, DG-75 and U-698 seemed to recover over time and displayed a similar viability as uninfected cells at 144 h.

### LOAd viruses replicate even in cells more resistant to killing

**Fig. 2:**
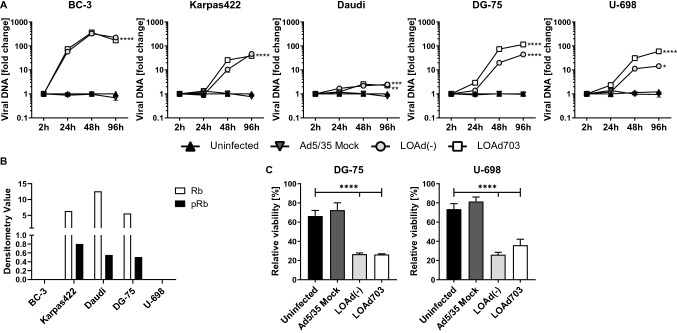
Investigation of LOAd replication in lymphoma cell lines. Lymphoma cells (BC-3, Karpas422, Daudi, DG-75 and U-698) were infected with LOAd viruses or replication-deficient Ad5/35 Mock (50 MOI), and viral DNA was isolated at 2, 24, 48 and 96 h post infection and quantified with primers detecting adenoviral DNA. Graphs display the representative of two independent experiments and shows mean fold change ± SD of triplicates wells of viral DNA compared to baseline (2 h post infection) (**a**). Bar graphs in (**b)** show the expression of retinoblastoma protein (Rb) and phosphorylated Rb (pRb) in the lymphoma cells as analyzed with Western blot. In (**c)**, DG-75 and U-698 cells were infected with LOAd viruses or replication-deficient Ad5/35 Mock (50 MOI). Supernatants from infected DG-75 and U-698 were collected 48 h post infection and added to plated Panc01 cells. Cell viability was analyzed with MTS viability assay 96 h later and is shown as percentage viability of control cells cultured with fresh medium (mean ± SD of technical triplicates). Statistical differences compared to uninfected control cells were analyzed (for A: at 96 h) with one-way ANOVA followed by Dunnett’s multiple comparisons test (*p < 0.05, **p < 0.01, ***p < 0.001, ****p < 0.0001)

To further investigate if BC-3 was indeed killed through oncolysis and if the decreased killing capability in the other cells was due to impaired viral replication, viral DNA was isolated over time and quantified with qPCR. Figure 2a displays the fold change of viral DNA over baseline. As expected, no replication of Mock virus was observed in any of the cells lines. LOAd viruses replicated best in BC-3 cells, which is in line with the highest viability reduction in these cells. Karpas422, DG-75 and U-698 displayed comparable viral DNA levels, even though only Karpas422 cells were efficiently killed. Infection of Daudi cells resulted in minimal viral replication. Replication could be inhibited by presence of unphosphorylated retinoblastoma protein (Rb), which would entrap E2F, a transcription factor that is required for initiation of LOAd replication. To investigate this, expression of Rb and phosphorylated Rb (pRb) was analyzed with Western blot (Fig. 2b). Rb was deleted in BC-3 and U-698 cells; hence, E2F remains free to enable viral replication. Karpas422, Daudi and DG-75 expressed Rb which was to some extent phosphorylated, and these cell lines should therefore in theory be able to replicate the LOAd viruses, but this assay does not detect possible Rb mutations that may affect E2F binding as well. The detected adenoviral DNA in the oncolysis-resistant DG-75 and U-698 cells may be explained by production of dysfunctional viral particles. To examine this, supernatants from infected DG-75 and U-698 cells were added to Panc01 cells, a pancreatic cancer cell line sensitive to LOAd infection [16]. Addition of supernatants from both LOAd-infected cell lines reduced the viability of Panc01 cells (Fig. 2c). Hence, even though DG-75 and U-698 were not efficiently killed by oncolysis, they were still capable of producing functional viruses that have oncolytic capacity in sensitive cells.

### LOAd infection induces transgene expression and a change in immune markers

**Fig. 3:**
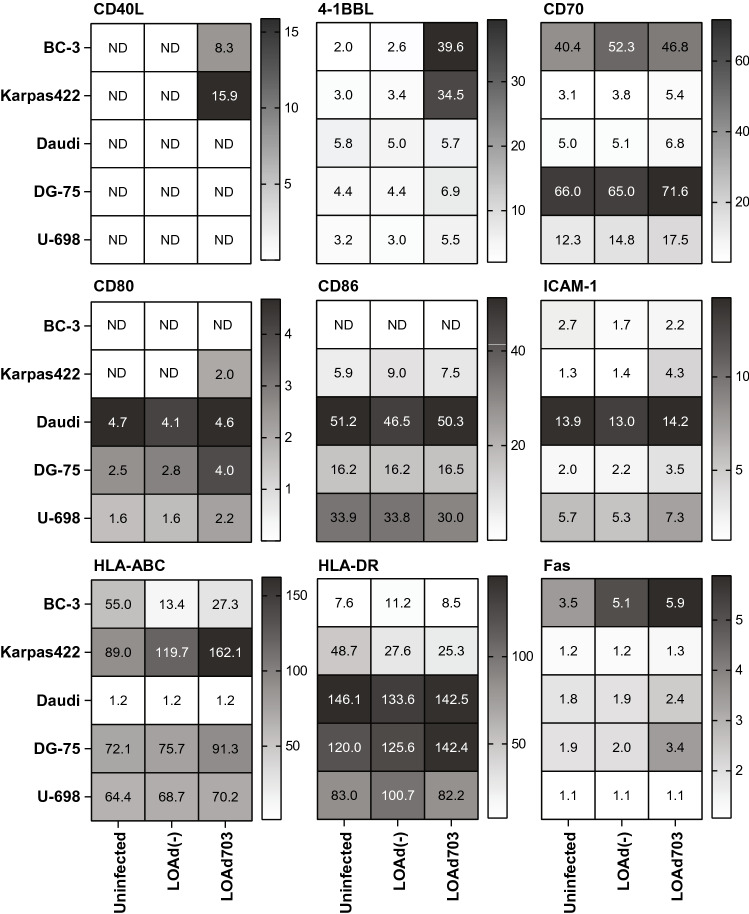
Expression of transgenes and immune markers in LOAd-infected lymphoma cell lines. Cells were infected with 100 MOI of LOAd(-) and LOAd703, cultured for 48 h and analyzed by flow cytometry for the expression of the transgenes CD40L and 4-1BBL, as well as immune markers CD70, CD80, CD86, ICAM-1, HLA-ABC, HLA-DR and Fas. Heat maps display the mean relative expression of two independent experiments (fold change of the geometric mean fluorescence intensity (MFI) of the marker over the MFI of the respective isotype control antibody). ND = not detected

LOAd virus infection and expression of the transgenes TMZ-CD40L and 4-1BBL by LOAd703 could potentially render the lymphoma cells more immunogenic, thereby making them better T cell targets. Expression of transgenes and immune markers was analyzed with flow cytometry 48 h post infection. Figure 3 shows the relative expression of the analyzed markers compared to isotype staining. CD40L could only be detected in LOAd703-infected BC-3 and Karpas422 cells. 4-1BBL was inherently expressed by all cell lines, but was upregulated upon LOAd703 infection. As B cells naturally express markers for antigen presentation and T-cell activation, we evaluated if LOAd infection changes these expression levels. The co-stimulatory molecule CD70 was slightly upregulated upon LOAd infection in all cells, and this was most prominent with transgene-encoding LOAd703. All cells, apart from BC-3, expressed the co-stimulatory molecules CD80 and CD86. Overall, CD80 was upregulated upon LOAd703 infection, whereas CD86 expression was rather stable. The adhesion molecule ICAM-1 was particularly upregulated upon LOAd703 infection. HLA-ABC was expressed highly in all cells apart from Daudi. A strong increase in expression was seen in LOAd703-infected Karpas422 and DG-75 cells. HLA-DR expression was highest in Daudi, DG-75 and U-698 and increased in LOAd703-infected DG-75. The death receptor Fas was minimally expressed in Karpas422 and U-698 cells and primarily increased in LOAd703-infected BC-3, Daudi and DG-75.

### Increased CAR T-cell activation with LOAd703-infected lymphoma cells

**Fig. 4:**
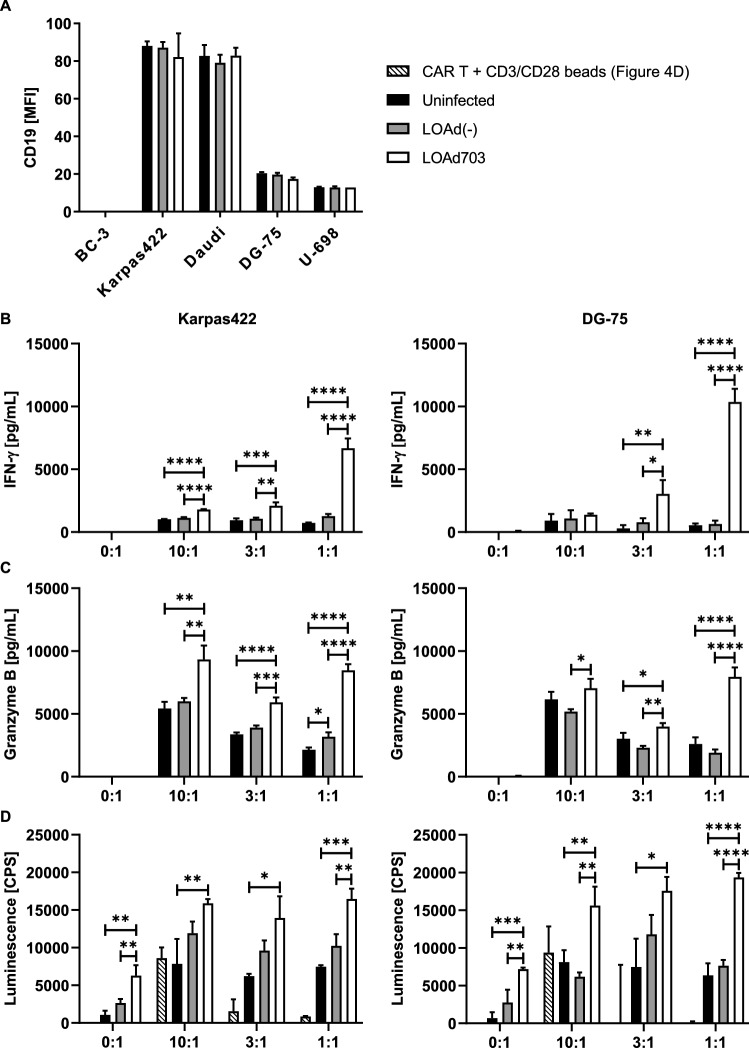
Combination of LOAd viruses with CAR T cells in a killing assay. CD19 expression was analyzed in LOAd-infected lymphoma cells by flow cytometry. Bar graphs in (**a)** show the relative geometric mean fluorescence intensity (rMFI) compared to isotype control staining. For (**b–d)**, CD19+ target cells Karpas422 and DG-75 cells were infected with 100 MOI of LOAd(-) or LOAd703 or left uninfected and cultured for 48 h. CAR T cells were thawed and cultured in 100 IU/ml IL-2 for 48 h. At 48 h, 5 × 10^4^ target cells were plated per well in triplicates in 96-well plates, and CAR T cells were added to achieve an effector/target cell ratio of 10:1–1:1. Forty-eight h after the co-culture setup, culture supernatants were taken and analyzed for release of IFN-γ (**b**), granzyme B (**c**) and lactate dehydrogenase (LDH) (D). Bar graphs show mean ± SD of three technical replicates. In (**d)**, background signal from medium and CAR T cells alone was subtracted from the LDH signal, and LDH release from CAR T cells stimulated with CD3/CD28 dynabeads (Thermo Fisher) is shown as control for activation-induced cell death of CAR T cells. Statistical differences comparing all groups to each other were analyzed with one-way ANOVA followed by Tukey’s multiple comparisons test (*p < 0.05, **p < 0.01, ***p < 0.001, ****p < 0.0001). In (**d)**, the CD3/CD28 bead control was excluded from statistical analysis

CAR T-cell therapy for B-cell lymphoma has not been as successful as for B-cell leukemia. Thus, novel strategies to improve responses are needed. As LOAd703 infection induces the expression of the immunostimulatory transgenes, as well as upregulates molecules that facilitate recognition and killing by T cells, LOAd703 treatment prior to CAR T-cell therapy may prime the lymphoma lesion and facilitate a more efficient CAR T-cell response. Infection of the lymphoma cell lines with LOAd viruses did not alter their CD19 expression levels (Fig. 4a); hence, a combination of both treatment modalities is feasible. We hypothesized that LOAd703-infected lymphoma cells, independently of their susceptibly to oncolysis, may lead to enhanced activation of CD19-targeting CAR T cells and increased lysis of the lymphoma cells. Therefore, we chose to use Karpas422 and DG-75 as an oncolysis sensitive and resistant cell line, respectively. The target cells were infected 48 h before co-culture with CAR T cells, and CAR T-cell activity was assessed 48 h after the start of co-culture. CAR T-cell activation was significantly augmented by stimulation with LOAd703-infected cells, as seen by both increased production of IFN-γ (Fig. 4b) and secretion of granzyme B (Fig. 4c). Target cell lysis was evaluated by analyzing LDH, which is released from dying cells, in culture supernatants (Fig. 4d). It should be noted that CAR T cells dying from activation-induced cell death may also release LDH. Hence, a control condition of CAR T cells stimulated with CD3/CD28 beads was added to the analysis. Indeed, activated CAR T cells alone released LDH, but the amount was minimal, except at the highest effector-to-target cell ratio. The level of LDH was increased in LOAd703-infected target cells compared with uninfected and LOAd(-)-infected target cells. CAR T cells also efficiently lysed target cells on their own even at the lowest CAR T cell-to-target cell ratio. However, the most efficient lysis was seen with LOAd703-infection combined with CAR T cells. Strikingly, the enhanced CAR T-cell activity was most evident in the lowest effector-to-target cell ratio and was consistent between the two different target cells used. The experiment was repeated by using two more CAR T-cell batches from different individuals with similar results (Supplementary Figure. 1).

### CAR T cells upregulate CD107a, while displaying reduced expression of PD-1 and TIM-3 in the presence of LOAd703-infected lymphoma cells

**Fig. 5:**
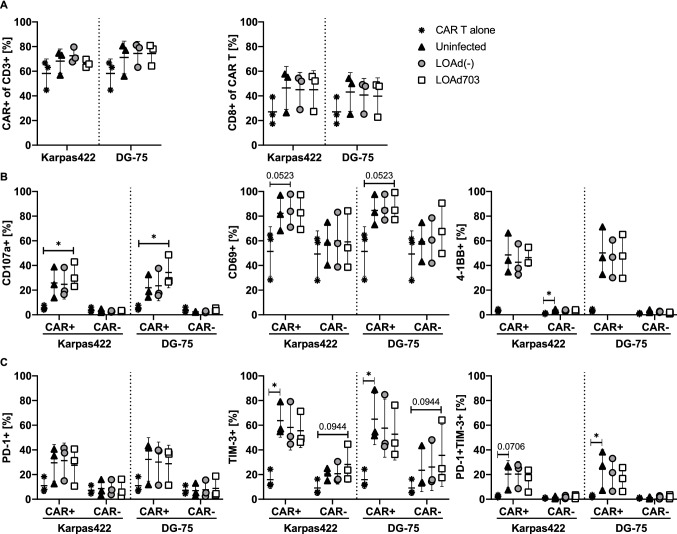
Functional status of CAR T cells analyzed with flow cytometry. The target cells Karpas422 and DG-75 cells were infected with LOAd(-) or LOAd703 (100 MOI) or left uninfected and cultured for 48 h. CAR T cells were thawed and cultured in 100 IU/ml IL-2 for 48 h. At 48 h, target and effector cells were cultured in a 1:1 ratio for 24 h. Cells were analyzed with flow cytometry for the expression of CAR and CD8 (**a**), as well as activation markers CD107a, CD69 and 4-1BB (**b**) and exhaustion markers PD-1 and TIM-3 (**c**). Graphs display the mean ± SD of the three independent experimental repeats with different CAR T cell batches. Statistical differences were analyzed with Kruskal–Wallis test followed by Dunn’s multiple comparison test comparing each group to CAR T cells alone (*p < 0.05)

To further study the functional status of the CAR T cells, we analyzed CAR T cells co-cultured with LOAd-infected target cells with flow cytometry. It should be noted that the three CAR T-cell batches used displayed varying expression levels of markers analyzed, and the number of replicates was low and mostly similar trends between the repeats are evaluated in Fig. 5. Nevertheless, statistical differences compared to the CAR T cell alone control group were evaluated with nonparametric testing. The percentage of CAR^+^ and the proportion of CD8^+^ cells within the CAR population was increased upon contact with the target cells in all co-cultures (Fig. 5a), but this was not statistical significant. Likewise, the activation markers CD107a, CD69 and 4-1BB were highly upregulated on CAR^+^ T cells in the presence of lymphoma cells in general, but CD107a expression was only significantly upregulated with LOAd703. CD107a and 4-1BB induction was specific for CAR^+^ T cells, whereas CD69 expression was also high on CAR^−^ T cells and slightly increased further with LOAd703-infected DG-75 (Fig. 5b), even though no statistical significant differences could be detected. The expression of PD-1 and TIM-3 was induced in the presence of target cells and overall higher for CAR^+^ T cells. Interestingly, co-culture with LOAd-infected target cells resulted in somewhat less TIM-3^+^ as well as double positive (PD-1^+^TIM-3^+^) CAR T cells since in particular TIM-3 was only significantly upregulated with uninfected target cells. Contrarily, CAR^−^ T cells appeared to display higher TIM-3 expression in LOAd703 samples (Fig. 5c). Based on that the release of granzyme B and IFN-γ in this experimental setup was consistent with what was previously shown for 48 h (data not shown); we anticipated overall more distinct phenotype changes when the CAR T cells were co-cultured with LOAd703-infected target cells with a clearly enhanced activation profile and reduced expression of exhaustion markers. However, this was not as apparent in this experimental setting, which may be due to the time point of analysis and dynamics of the marker expression.

### LOAd703-infected lymphoma cells secrete chemokines and attract CAR T cells

**Fig. 6:**
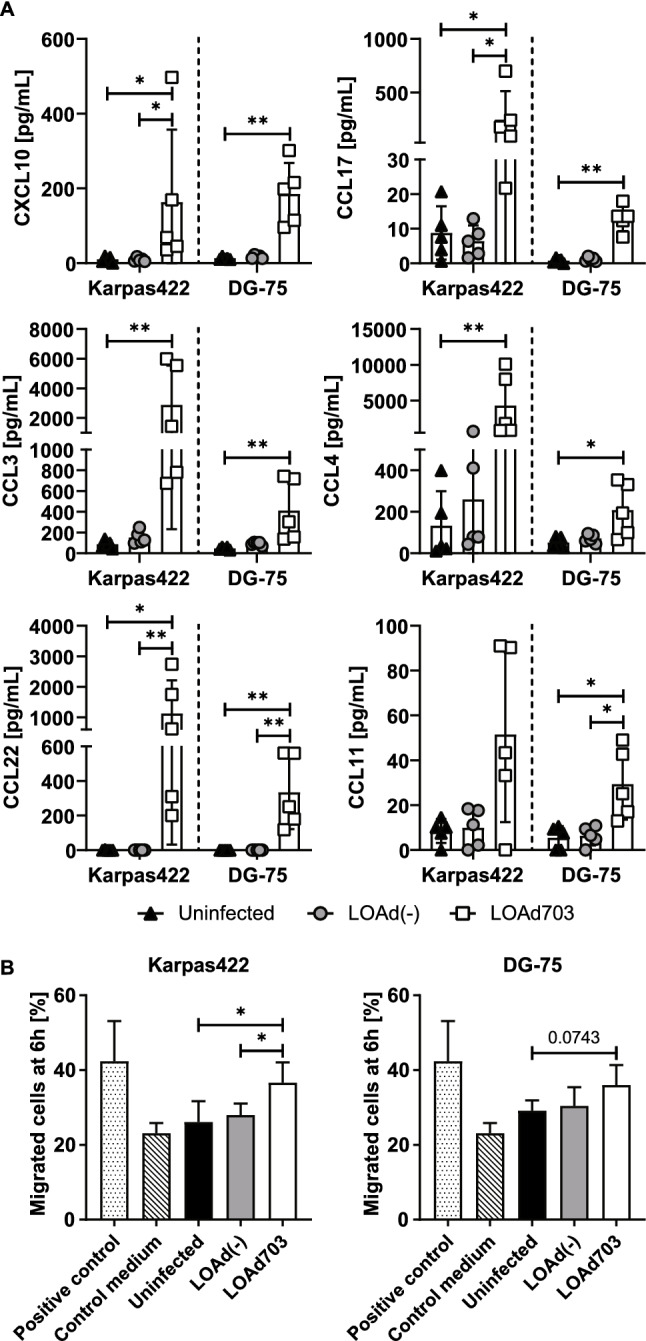
CAR T-cell migration assay and chemokine analysis in lymphoma supernatants. Karpas422 and DG-75 cells were infected with LOAd(-) or LOAd703 (100 MOI) or left uninfected and cultured for 48 h before culture supernatants were collected and analyzed for chemokine expression with V-PLEX Chemokine Panel 1 (Meso Scale Diagnostics). Bar graphs show mean ± SD of five biological replicates (**a**). To assess CAR T-cell migration toward these culture supernatants, 600 µl of the culture supernatants were loaded in the lower chamber of a transwell. As positive control, medium supplemented with 250 ng/ml CXCL10 was used. 1 × 10^6^ CAR T cells were placed on the upper chamber, and migrated cells were counted in the lower chamber 6 h later. Bar graphs show mean ± SD of technical duplicates of three independent experimental repeats (**b**). Statistical differences were determined with Kruskal–Wallis test followed by Dunn’s multiple comparison test comparing all groups to each other, but in B the positive and negative control was removed from statistical analysis (*p < 0.05, **p < 0.01)

We hypothesized that priming of lymphoma lesions with LOAd viruses may facilitate CAR T-cell recruitment by the induction of chemokines. Supernatants from infected lymphoma cell lines were analyzed for chemokine levels with a multiplex assay (Fig. 6a). Infection with LOAd703 significantly enhanced the secretion of various chemokines in Karpas422 and DG-75 cells. These included CXCL10, CCL17, CCL3, CCL4, CCL22 and CCL11. Chemokine expression in the other investigated cell lines is shown in Supplementary Fig. 2, and interestingly, LOAd703 also induced high chemokine secretion in the otherwise largely unaffected Daudi cells. To evaluate if the CAR T cells would indeed migrate more toward these supernatants, a transwell migration assay was set up with supernatants from Karpas422 and DG-75 cells. In agreement with the enhanced chemokine production, CAR T-cell migration was significantly higher toward supernatants from LOAd703-infected Karpas422 and DG-75 cells than toward supernatants from uninfected lymphoma cells (Fig. 6b).

### Combination of LOAd703 with CAR T cells in a xenograft model

**Fig. 7:**
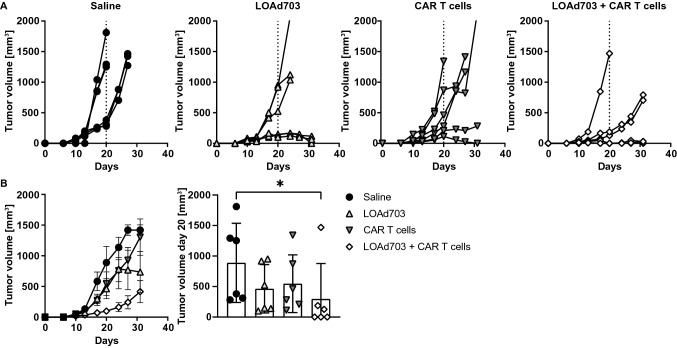
Combination of LOAd703 with CAR T cells in an in vivo xenograft model. DG-75 cells were injected subcutaneously in female immunodeficient BALB/c nude mice (*n* = 7 per group). Treatments were initiated ten days after tumor injection and mice received in total 6 intratumoral treatments with LOAd703 (1 × 10^9^ FFU/mouse) or saline as control. At the second treatment time point (day 13), CAR T cells (3.3 × 10^6^ cells/mouse) were administrated intravenously. In (**a)**, the tumor growth curves of each individual mouse are displayed. Mice with no tumor take (*n* = 1 for saline and LOAd703 group) and that did not receive the full intravenous CAR T cell dose due to technical difficulties (*n* = 1 for CAR T cell and LOAd703 + CAR T cell group) were excluded from the experiment. In (**b)**, the mean tumor volume curves ± SEM are displayed and the bar graphs show the mean ± SD tumor volume at day 20, when no mice had been sacrificed yet, which allows for direct comparison of the mean tumor volumes. Statistical differences were determined with Kruskal–Wallis test followed by Dunn’s multiple comparison test comparing each group to the saline control (*p < 0.05)

As the LOAd703 virus needs human CD46 for infection, encodes human transgenes and cannot replicate in mice, the combination therapy cannot be evaluated in immunocompetent syngeneic mice models. However, we sought to investigate if LOAd703 can support CAR T cells in a xenograft model by debulking the tumor mass and expression of the transgenes. Subcutaneously established DG-75 tumors were injected with LOAd703 or saline control for a total of six treatments. At the second treatment time point (day 13), CAR T cells were administered intravenously. Figure 7a displays the tumor growth curves of each individual mice, and tumor growth was delayed in 3/6 and 2/6 mice (50% and 33% response rate) with single treatments of LOAd703 and CAR T cells, respectively. In contrast, the combination treatment delayed tumor growth in 5/6 mice (83% response rate). The average tumor volume was compared at day 20 before any mice were killed due to too large tumors, and only the combination treatment showed a significant reduction in tumor volume compared to saline control (Fig. 7b). Hence, LOAd703 treatment could facilitate CAR T cell therapy in an in vivo setting, which is in agreement with our in vitro results.

## Discussion

CAR T-cell therapy is gaining ground in lymphoma treatment, but there is a need to increase response rates and decrease post-therapy relapses. The main hurdle for CAR T-cell therapy in lymphoma is thought to be the harsh environment within these solid lesions, with presence of both physical barriers and immunosuppressive cells/factors [6]. Thus, it is appealing to combine CAR T-cell therapy with treatments that stimulate the TME and thereby support CAR T-cell activation. Gene engineering of the tumor and its stroma with oncolytic viruses is an interesting option [18, 19]. The oncolytic virus used herein, LOAd703 (delolimogene mupadenorepvec), delivers genes that activate antigen-presenting cells and T cells and is already being evaluated in pancreatic, biliary, colorectal and ovarian cancer as well as in melanoma for its ability to increase immune responses in the TME.

Adenoviruses have not been widely utilized in hematological malignancies as the most commonly used serotype five infects cells via the coxsackie and adenovirus receptor, which is absent or expressed at low levels in hematologic cells [20]. LOAd703 is a serotype chimera and uses the serotype 35 fiber for infection via CD46 [21]. Chimeric Ad5/35 vectors infect normal cells and malignant B cells at better rates than Ad5 [21–23]. All herein investigated lymphoma cells expressed CD46 and had a dysregulated retinoblastoma pathway, which should enable LOAd infection and replication. Nevertheless, only BC-3 and Karpas422 were killed efficiently by LOAd infection while Daudi, DG-75 and U-698 were killed to a low degree only at high viral load. In DG-75 and U-698, the resistance to oncolysis appeared despite virus replication and release of functional viruses. It should be noted that the readout of the MTS viability assay is metabolic activity; thus, an expected reduction in signal by dying cells could be masked by a fraction of the cells that get stimulated by LOAd infection, which could be the case for DG-75 and U-698. Also, Karpas422 cells appeared to be less efficiently killed by transgene expressing LOAd703 virus, which could likewise be explained by a stimulation of the lymphoma cells through CD40L/CD40 signaling. Furthermore, Zhang et al. noted that some lymphoma cells were able to proliferate normally post Ad5 infection, while continuously replicating the viral DNA [24]. In contrast, Daudi displayed very low replication, did not express the transgenes and had the lowest CD46 expression, meaning that limited viral entry may explain the poor virus replication and reduced oncolysis. However, Drouin et al. found that the transduction efficiency of Ad5/35 viruses in malignant B cells did not correlate to CD46 expression. Instead, they demonstrated different intracellular localization of Ad5/35 viruses dependent on the permissiveness of the cells, and in non-permissive Daudi cells, viruses were confined to late endosomes and degraded in lysosomes [25]. The alternative trafficking routes were suggested to be regulated by the differentiation status of the cell type with plasma cells being most permissive. Likewise, we have previously shown efficient oncolysis of multiple myeloma cell lines using LOAd viruses [26].

Despite a partial resistance to oncolysis, most LOAd703-infected lymphoma lines expressed the CD40L/4-1BBL transgenes and upregulated co-stimulatory, adhesion and MHC molecules, which together may enhance anti-lymphoma T-cell responses. The lack of transgene expression in Daudi cells is likely due to the limited viral replication in this cell line, whereas the absent CD40L expression in DG-75 and U-698 may be caused by their higher CD40 expression compared to Karpas422 (data not shown) as the resulting CD40L/CD40 interaction may mask the binding site for the detection antibody. In abnormal B cells, CD40/CD40L signaling has been suggested to improve antigen presentation and generation of antigen-specific cytotoxic T cells [27]. Murine A20 B-cell lymphoma cells expressing CD40L have been shown to upregulate CD80, CD86, ICAM-1, Fas and MHC molecules. Mice vaccinated with these cells induced an antigen-specific immune response against the parental cells [28–30]. Further, A20 expressing 4-1BBL in combination with CD80 and CD86 have been observed to be highly immunogenic, thereby inducing durable anti-tumor responses [31]. Likewise, CD40L gene transfer in B-cell lymphoma cells from patients resulted in enhanced expression of CD80 and CD86 [32] and generation of tumor-specific T-cell response in vitro [32, 33]. Our results are in agreement with these observations and indicate that LOAd703 augmented the immunogenicity of most lymphoma cells. BC-3 cells appeared to have a distinct phenotype with no expression of CD80 and CD86, which is likely caused by their inherent infection with Kaposi's sarcoma-associated herpesvirus [34–36]. However, BC-3 displayed the highest upregulation of the death receptor Fas upon LOAd infection, and this might mediate Fas-FasL-dependent killing by T cells, which is a crucial pathway in the immune surveillance of B-cell lymphomas [37]. In addition to direct effects on the lymphoma cells, most cell lines did produce functional viruses, and in an in vivo situation, these released viruses would spread and infect surrounding stroma cells, where they would induce transgene expression independently of viral replication.

Further, priming lymphoma lesions with oncolytic viruses is an intriguing approach to potentiate CAR T-cell therapy. The oncolytic process may debulk the tumor, and the resulting inflammatory response may counteract the immunosuppressive microenvironment, thereby facilitating CAR T-cell recruitment. Moreover, virus-encoded transgenes may directly enhance T-cell function [18]. Indeed, CAR T-cell activation was stronger in response to LOAd703-infected target cells as observed by a higher production of IFN-γ, enhanced degranulation (increased CD107a surface expression) and release of granzyme B together with an increase in cytolytic activity (higher LDH release). In agreement, we noted slightly less PD-1^+^TIM-3^+^ CAR T cells in co-cultures with LOAd703-infected target cells. In B-cell lymphoma, intratumoral T cells co-expressing PD-1 and TIM-3 have been shown to be exhausted [38] and, thus, LOAd703 infection in tumors might lead to less exhausted T cells with higher cytotoxic function. Interestingly, the CAR T-cell response was augmented most strongly in co-cultures with lower effector-to-target cell ratio, which may be due to prolonged presence of the tumor cells that are functioning as stimulators in this assay. Importantly, both a cell line susceptible to and a cell line more resistant to oncolysis equally boosted CAR T-cell function after LOAd703 infection. Thus, the combination treatment may be beneficial even for oncolysis-resistant lymphoma tumor subtypes as the oncolytic function itself does not seem crucial for the enhanced CAR T-cell activity, and the stimulatory effect appears to be largely driven by the transgene expression induced by LOAd703.

LOAd703-infected tumor cells released more chemokines involved in T-cell recruitment, and CAR T cells migrated to a greater extent toward supernatants from these cells in vitro. CXCL10 expression is crucial for the recruitment of effector T cells and natural killer cells [39]. CCL17 and CCL22 induced by CD40 stimulation on B-cell leukemia cells have been shown to attract CCR4^+^ tumor-specific T cells [40]. CCL3 and CCL4 are involved in the recruitment of T cells as well as dendritic cells [41–43], and vaccination strategies containing CCL3 could establish immune response against A20 [44]. Thus, LOAd703 infection in lymphoma lesions may lead to improved recruitment of CAR T cells, but also of other immune cells such as dendritic cells, important for the induction of anti-tumor immunity. These immune cells may further stimulate the CAR T cells, but also bystander T cells with natural immunity to lymphoma antigens. We observed some indication of bystander T cell activation as CAR^−^ cells seemed to upregulate CD69 and TIM-3 upon co-culture with LOAd703-infected lymphoma cells. This would be beneficial in case CD19 negative tumor clones appeared, which is common during CD19-directed CAR T-cell therapy [45, 46]. It would be of interest to study the effect of the combination of LOAd703 and CAR T cells in a syngeneic immunocompetent murine in vivo model in order to be able to also investigate the effect on other immune cells and in an immunosuppressive tumor microenvironment as well as in a systemic setting. As the transgene expression is independent of viral replication and driven by a CMV promoter, infected bystander immune and stromal cells in the tumor microenvironment could also express the transgenes, which would likely further promote anti-tumor responses. For example, we have shown in vitro that LOAd703 infection of stromal cells leads to upregulation of adhesion molecules and chemokines crucial for lymphocyte trafficking [7], which may further promote CAR T cell infiltration. It should be noted that the transgenes expression in non-tumor cells would be transient and would only occur in the injected tumor microenvironment, meaning that adverse effects caused by a systemic overstimulation of the immune system are unlikely. Unfortunately, studies in immunocompetent mouse models are difficult as we used human CAR T cells derived from patient material in this study. Moreover, LOAd viruses cannot efficiently infect murine cells due to the missing CD46 receptor in the murine system and adenoviruses cannot replicate in mice [47], which would preclude any evaluations of the oncolytic effects even if LOAd viruses expressing murine equivalent transgenes and murine CAR T cells could be used. However, we could demonstrate in a subcutaneously established xenograft tumor model that intratumoral LOAd703 treatments could promote CAR T cell responses leading to enhanced tumor control also in an in vivo setting.

## Conclusion

Serotype 5/35 oncolytic adenoviruses within the LOAd platform were able to infect and replicate in most B-cell lymphoma cell lines. LOAd703 armed with TMZ-CD40L and 4-1BBL induced transgene expression and an immunogenic phenotype by upregulating molecules that facilitate immune surveillance. Even if different types of B-cell lymphoma may be partially resistant to oncolysis, the collective mechanisms-of-action may still support CAR T-cell combination therapy as shown by enhanced cytotoxic T-cell function and migration by both oncolysis-susceptible and oncolysis-resistant LOAd-infected lymphoma cells. Hence, combining LOAd703 and CAR T-cell therapy is an intriguing approach in B-cell lymphoma and trials are warranted.

### Supplementary Information

Below is the link to the electronic supplementary material.

Supplementary file 1 (PDF 704 kb)

## Data Availability

The datasets used and/or analyzed during the current study are available from the corresponding author on reasonable request.

## Abbreviations

CAR: Chimeric antigen receptor
FBS: Fetal bovine serum
LOAd: Lokon oncolytic adenovirus
LDH: Lactate dehydrogenase
MFI: Geometric mean fluorescence intensity
MOI: Multiplicity of infection
pRb: Phosphorylated retinoblastoma protein
Rb: Retinoblastoma protein
rMFI: Relative geometric mean fluorescence intensity
TMZ-CD40L: Trimerized membrane-bound CD40L
