# Supplementary material for: Boosting CAR T-cell responses in lymphoma by simultaneous targeting of CD40/4-1BB using oncolytic viral gene therapy
Source: Cancer Immunol Immunother. 2021 Mar 5;70(10):2851–65. doi: 10.1007/s00262-021-02895-7 (PMC8423656; doi:10.1007/s00262-021-02895-7)
Supplement: Supplementary file 1 — (PDF 704 kb) [file 262_2021_2895_MOESM1_ESM.pdf]

Supplementary Figure 1

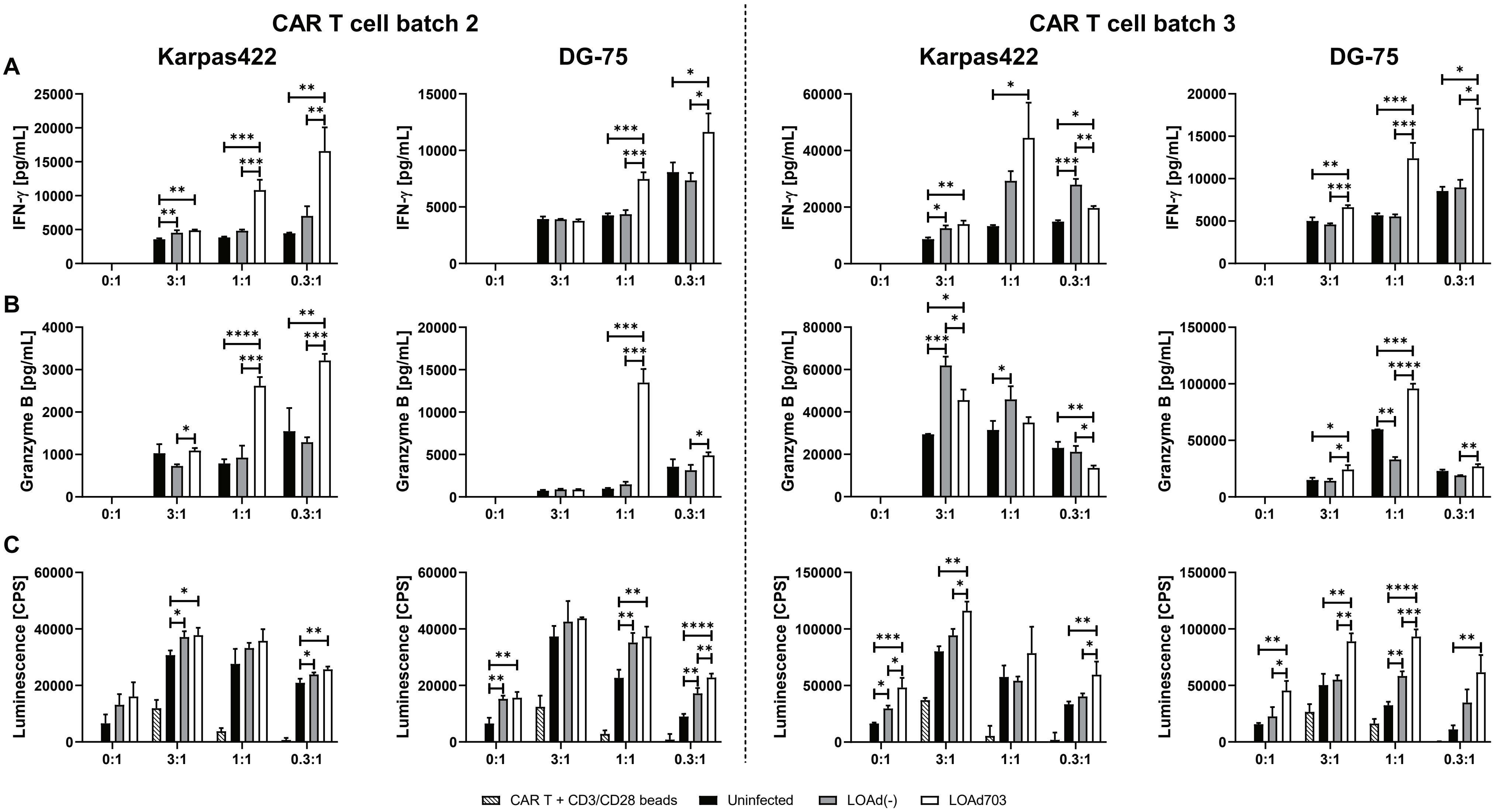

Supplementary Figure 1: CAR T cell killing assay repeats The target cells Karpas422 and DG-75 cells were infected with LOAd(-), LOAd703 (100 MOI) or left uninfected and cultured for 48 hours. CAR T cells were thawed and cultured in 100 IU/ml IL-2 for 48 hours. At 48 hours, both target and effector cells were harvested and counted. 5×10^4 target cells were plated per well in triplicates in 96-well plates and CAR T cells were added to achieve an effector:target cell ratio of 3:1-0.3:1. 48 hours after the co-culture set-up, cell culture supernatants were taken and analyzed for release of IFN-γ (A), Granzyme B (B) and lactate dehydrogenase (LDH) (C). Bar graphs show mean ± SD of three technical replicates. In C, background signal from medium and CAR T cells alone was subtracted from LDH signals and LDH release from CAR T cells stimulated with CD3/CD28 beads is shown as control for activation-induced cell death of CAR T cells. Statistical differences comparing all groups to each other were analyzed with one-way ANOVA followed by Tukey's multiple comparisons test (*$p<0.05$, **$p<0.01$, ***$p<0.001$, ****$p<0.0001$). In C, the CD3/CD28 bead control was excluded from statistical analysis.

Supplementary Figure 2

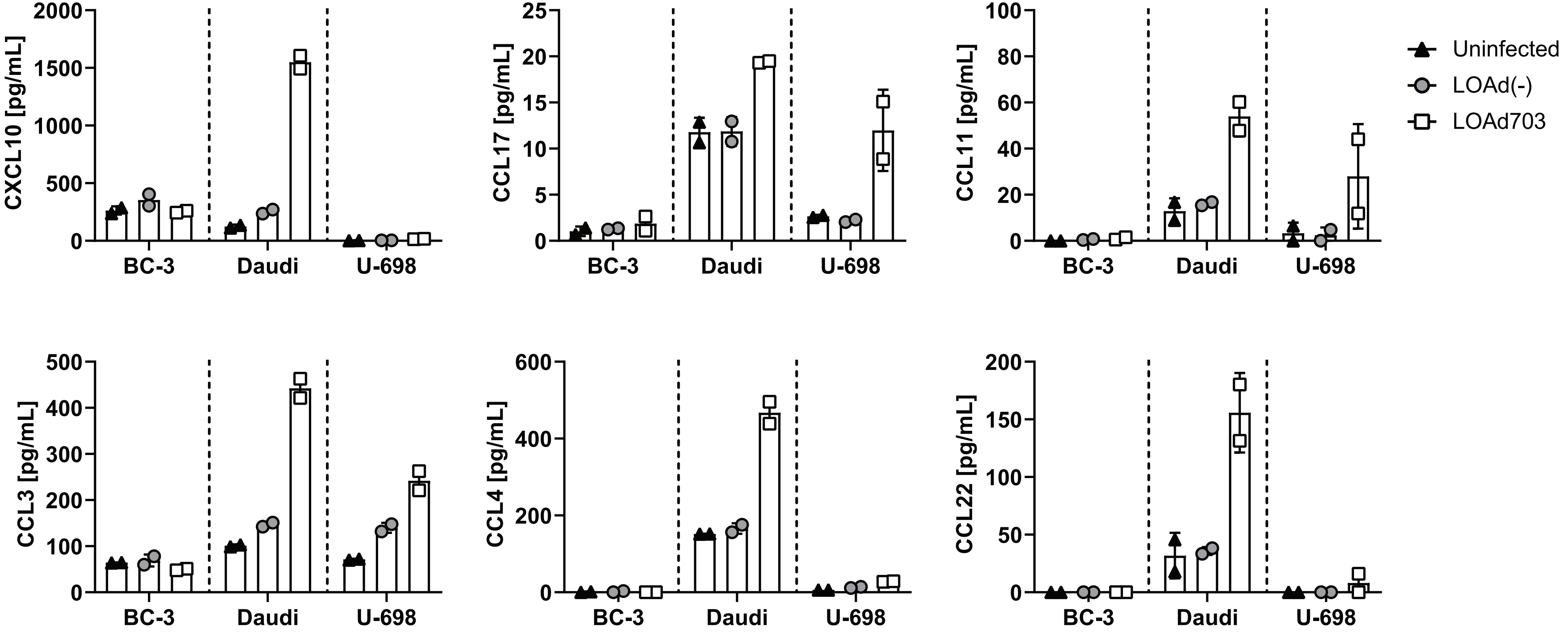

Supplementary Figure 2: Chemokines expressed by BC-3, Daudi and U-698 cells BC-3, Daudi and U-698 cells were infected with LOAd(-), LOAd703 (100 MOI) or left uninfected and cultured for 48 hours. Cell culture supernatants were taken and analyzed for chemokine expression with V-PLEX Chemokine Panel 1 (Meso Scale Diagnostics). Bar graphs show mean ± SD of two biological replicates.
